# Arthrogryposis multiplex congenital in a child manifesting phenotypic features resembling dysosteosclerosis/osteosclerosis malformation complex; 3DCT scan analysis of the skull base

**DOI:** 10.1186/1757-1626-1-56

**Published:** 2008-07-23

**Authors:** Ali Al Kaissi, Georg Kalchhauser, Franz Grill, Klaus Klaushofer

**Affiliations:** 1 Ludwig Boltzmann Institute of Osteology, at the Hanusch Hospital of WGKK, Vienna, Austria; 2 AUVA Trauma Centre Meidling, 4th Medical Department, Hanusch Hospital, Vienna, Austria; 3 Hanusch Hospital, Department of Radiology, Vienna, Austria; 4 Orthopaedic Hospital of Speising, Paediatric Department, Vienna, Austria

## Abstract

**Introduction:**

A boy presented with arthrogryposis multiplex congenita (AMC) associated with severe central nervous system dysfunction. The clinical history and the distinctive radiographic/tomographic features were consistent but not completely diagnostic for dysosteosclerosis.

**Case presentation:**

A 5-year-old boy from a consanguineous family in Austria was born with arthrogryposis multiplex congenita in connection with central nervous system dysfunction. Recently he was referred to the orthopaedic department for further clinical assessment. Radiographic documentation showed significant sclerosis and thickening at the skull base with further extension to involve the craniocervical junction. Spinal radiographs showed platyspondyly of the thoracic vertebral bodies associated with widening of the intervertebral spaces. Long bones were not sclerotic as usually seen in the classical dysosteosclerosis phenotype. It is highly likely that long-term immobilization because of arthrogryposis multiplex congenita was the main reason behind this. 3 DCT scans showed significant hypertrophy of the clivus. The latter occupied the major space of the skull base. The overall radiographic and scanning images were compatible but not fully diagnostic with dysosteosclerosis/osteosclerosis malformation complex.

**Conclusion:**

The skull base malformation complex in patients with dysosteosclerosis/osteosclerosis requires careful evaluation. 3DCT scanning of the skull base and the vertebrae could be useful tools for early recognition of the pathophysiological mechanism in patients with dysosteosclerosis/osteosclerosis/multiple contractures spectrum Previously, radiographs only have assessed the skull base pathology in patients with dysosteosclerosis, here we further characterize the pathology via 3DCT scan. Our patient illustrates extensive sclerosis of the skull base, associated with extremely hypertrophied clivus. The latter occupied the whole space of the skull base and the craniocervical junction.

We review the pertinent literature, discuss the differential diagnosis and suggest that our case was consistent but not fully compatible with dysosteosclerosis. We believe that our present patient represents either a novel type of dysosteosclerosis or a variant of osteosclerosis/arthrogryposis spectrum from a consanguineous family in Austria.

## Introduction

Arthrogryposis multiplex is a symptom complex rather than a diagnosis; it has various causes and appears as many different recognizable syndromes [1-3]. The disorder is not a single entity but may be due to a variety of prenatal conditions, particularly neuromuscular disorders such as congenital muscular dystrophy, and congenital dystrophia myotonica [4,5]. In the absence of generalized neuromuscular disease, the search for other reasons is mandatory.

Osteosclerosis in connection with severe central nervous system involvement has been well documented in the literature [6-8]. Spranger et al [8] had used a term of dysosteosclerosis to distinguish a syndrome chiefly characterised by osteosclerosis and platyspondyly. Previously, radiographic documentation was the only applied modality [9-11]. We introduced 3DCT scanning to further understand the skull base/vertebral malformation complex. Numerous measurements have been applied to confirm the correlation between prenatal onset skull base sclerosis and the development of serious neurological deficits [12]. Parents are first degree related and this supports autosomal recessive pattern of inheritance. The skull base in our present patient has been analyzed via 3DCT scan.

## Case presentations

**Figure 1:**
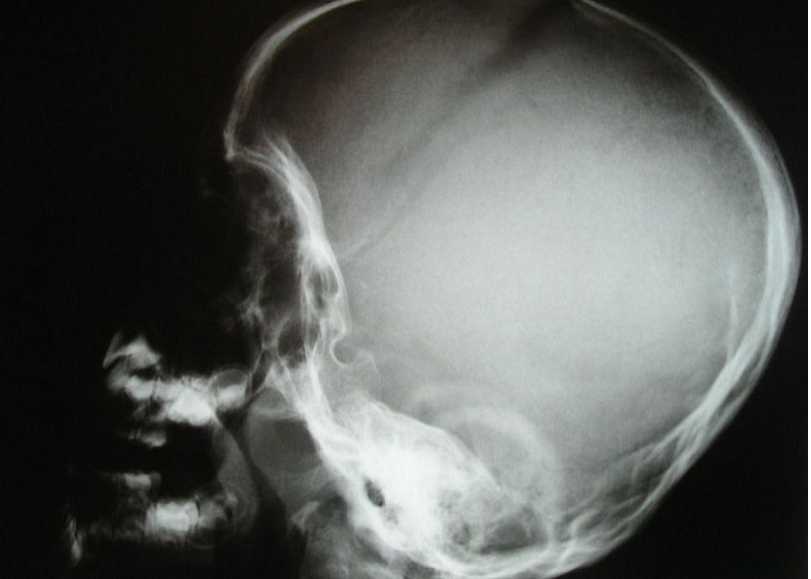
**Lateral skull radiograph shows a deformational brachycephalic skull.** It is most likely resulted from prolonged lying on the back, however, craniosynostosis was ruled out by clinical and radiographic means. There was thickening and sclerosis of the base of the skull, but a normal density and thickness of the vault was present. The anterior fontanelle is still opened.

**Figure 2:**
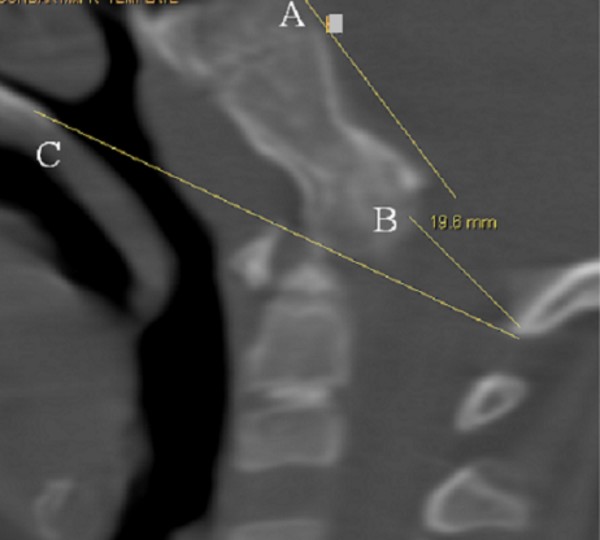
**3 D sagittal CT scan showed a hypertrophied clivus. **Line (A) is the Wachenheim clivus line (a method to evaluate and assess craniocervical junction abnormality), for which a line drawn along the posterior aspect of the clivus toward the odontoid process. In normal individuals the line must intersect/ tangential to the odontoid. In our present patient the line is remarkably deviating, outlining the existence of significant craniocervical abnormality. Line (B) is McRae’s line, which is essentially a measurement across the foramen magnum, and is drawn from the tip of clivus (basion) to opisthion (posterior margin of foramen magnum). McRae’s line is generally longer than 30 mm in normal individuals. A diameter of less than 25 mm is almost always associated with neurological abnormalities. In our patient McRae’s line showed a length of 19.6 mm. Line (C) is McGregor’s line, which is drawn from the posterior edge of the hard palate to the most caudal point of the occipital curve. An odontoid tip extending more than 4.5 mm above this line is considered abnormal. In our patient the hyperplastic clivus (the basion) is reversing the mechanism by extending downwards and joining the cephalad part of the odontoid.

**Figure 3:**
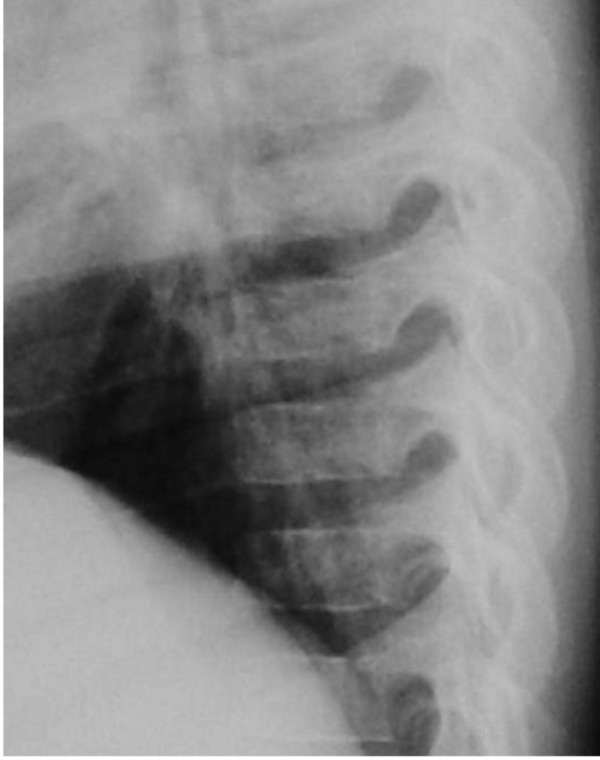
Lateral spine radiograph shows platyspondyly of the thoracic vertebral bodies associated with widening of the intervertebral spaces.

**Figure 4:**
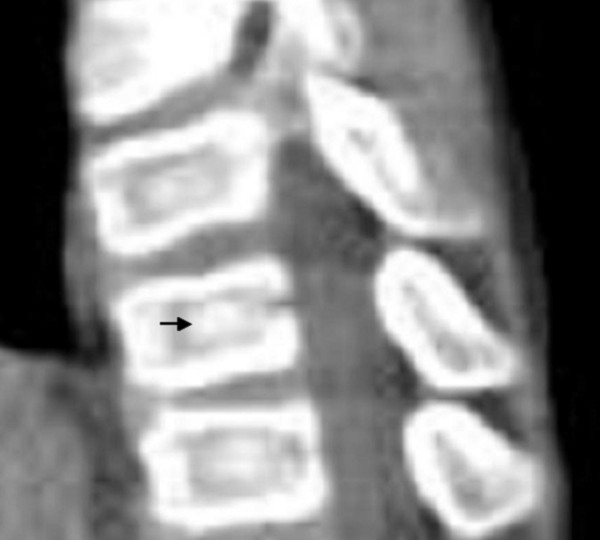
Sagittal 3DCT scan of the spine shows islands of increased sclerosis in areas of relative radiolucencies within the vertebral bodies.

**Figure 5:**
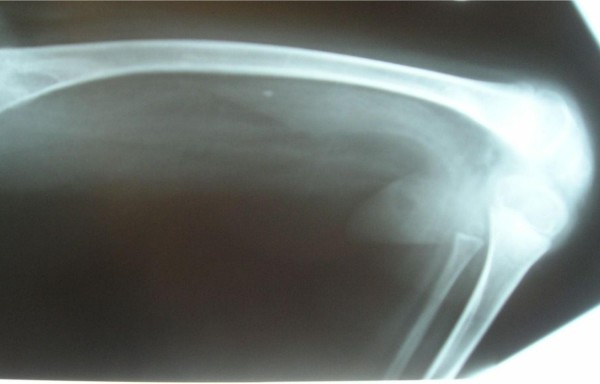
Anteroposterior radiograph of the lower limb showed significant fixed flexion deformity of 95° associated with minimal sclerosis around the metaphysis.

The present patient was referred to the orthopaedic department because of arthrogryposis multiplex. He was the first-born child of first degree-related parents (cousins). At birth his growth parameters were around the 25^th ^percentile. The mother, 28-years-old-gravida 2 abortus 0, her last trimester gestation was complicated by polyhdramnios. The father was a healthy, 33-years-old first degree relative. Family history was un-remarkable. The child was born with significant multiple contractures and a neurologic evaluation suggested severe psychomotor retardation associated with seizures. MRI scanning at the age of 9 months showed diffuse cortical dysplasia associated with retarded white matter meylination. The meylination pattern was consistent with an age of approximately 4 months according to the templates of MR images of normal brain development given by Barkovich [13]. He also had been admitted to intensive care for bouts of respiratory distress. Peripheral blood tests showed decreased hemoglobin and hematocrite levels. All serologic tests for congenital or acquired viral infections were negative. His subsequent course of development has been of severe retardation. Clinical examination showed growth around the 25^th ^percentile. Craniofacially he has a relative frontal bossing, slightly coarse facial features, mild hypertelorism, thin upper and lower lips, rather wide nasal tip, and thick eyebrows. There is brachycephaly; it is more likely resulted from prolonged lying on the back. Craniosynostosis, however, was ruled out. Extensive skull base sclerosis (fig 1) resulted in the development of massive craniocervical malformation complex. 3 D sagittal CT scan showed a hypertrophied clivus, Line (A) is the Wachenheim clivus line (a method to evaluate and assess craniocervical junction abnormality), for which a line drawn along the posterior aspect of the clivus toward the odontoid process. In normal individuals the line must intersect/ tangential to the odontoid. In our present patient the line is remarkably deviating, outlining the existence of significant craniocervical abnormality. Line (B) is McRae’s line, which is essentially a measurement across the foramen magnum, and is drawn from the tip of clivus (basion) to opisthion (posterior margin of foramen magnum). McRae’s line is generally longer than 30 mm in normal individuals. A diameter of less than 25 mm is almost always associated with neurological abnormalities. In our patient McRae’s line showed a length of 19.6 mm. Line (C) is McGregor’s line, which is drawn from the posterior edge of the hard palate to the most caudal point of the occipital curve. An odontoid tip extending more than 4.5 mm above this line is considered abnormal. In our patient the hyperplastic clivus (the basion) is reversing the mechanism by extending downwards and joining the cephalad part of the odontoid (fig 2). The spine showed relative stiffness, platyspondyly of the thoracic vertebral bodies associated with widening of the intervertebral spaces (fig 3). Sagittal 3DCT scan of the spine showed islands of increased sclerosis in areas of relative radiolucencies within the vertebral bodies (fig 4). Fixed flexion deformity along the knees was apparent. The sclerotic process within the long bones was not as usually seen in the classical dysosteosclerosis phenotype (fig 5). It is highly likely that long-term immobilization because of arthrogryposis multiplex congenita was the main reason behind this.

No history of fractures. The laboratory findings showed normal TSH/T4, a negative Guthrie test and normal karyotype.

## Discussion

Arthrogryposis multiplex is a term used to describe multiple congenital contractures; it is not a specific diagnosis. There have been a number of studies concerning the syndromic and non-syndromic entities in connection with arthrogryposis multiplex congenita (AMC) [1-5]. The larger and more heterogeneous series of 74 cases of AMC reviewed by Banker [2], showed 69 cases secondary to severe neurogenic disorders and 5 of myopathic type. Specific syndromes recognized included those of Pierre-Robin, Möbius, Meckel-Gruber, Arnold Chiari, Zellweger, Werding Hofman and prune belly. Dysgenesis of the central nervous system was found in 24 cases, with chromosomal abnormality in eight.

Hageman et al [3] made a prospective clinical study of 75 patients with multiple congenital contractures and achieved a nosological or syndrome diagnosis in 61 cases using clinical, laboratory and neurological data (there were 23 cases with perinatal death). The 21 cerebral disorders in this series included seven cases of the Pena-Shokier syndrome. A relationship of Pena-Shokeir syndrome/fetal hypokinesia deformation sequence and the development of arthrogryposis had been documented by Yfantis at al [14]. Significant developmental delay or regression, unexplained seizures, retinopathy, radiological brain changes as well as radiographic features of osteopetrosis might encompasses a heterogeneous group of disorders such as osteopetrosis and neuronal storage disease, and neuraxial dystrophy [15]. In infancy, the majority of cases of dysosteosclerosis are diagnosed as severe infantile autosomal recessive form of osteopetrosis. But, children with congenital osteopetrosis should not have central nervous system involvement and arthrogryposis is not a feature. The autosomal dominant form of osteopetrosis is far less severe than the autosomal recessive form. In the original form described by Albers-Schonberg [16] (now designated type II) clinical problems include bone fragility, osteomyelitis, dental abscesses and mild anemia.

Syndromes with osteosclerosis/central nervous system dysfunction were considered in the differential diagnosis [6-9]. None of the above-mentioned entities seems likely in the present patient and 3DCT scanning has not been a modality of investigation. Consanguinity of the parents may point to an autosomal recessive pattern of inheritance.

Our present patient showed the main clinico-radiographic features of dysosteosclerosis, namely prenatal severe central nervous system dysfunction, extensive sclerosis of the skull base and platyspondyly. Individuals with AMC require vigorous therapy and surgical intervention. While there is no cure, symptoms and deformities may still be alleviated with various methods due to multiple contractures and weakness. Orthopedic surgery is usually needed to correct severely affected joints and limbs and symptoms such as the multiple fixed flexion deformities as seen in our patient.

## Conclusion

Dysosteosclerosis is a serious neurodegenerative illness accompanied by unusual skeletal changes mainly diagnosed on skeletal survey. In addition to the neurological deterioration, the children have delayed milestones and are probably retarded from the beginning. Previously, radiographic documentation was the only modality to assess the skull-base pathology in patients with osteosclerosis/dysosteosclerosis complex. Here, we introduced 3 DCT scanning to further understand the etiology behind the malformation complex. We believe that our present patient represents either a novel type of dysosteosclerosis or a variant of osteosclerosis/arthrogryposis spectrum from a consanguineous family in Austria.

## Abbreviations

AMC: Arthrogryposis multiplex congenita; 3DCT: Three-Dimensional Computerized Tomography; SD: Standard deviation

## Competing interests

The authors declare that they have no competing interests.

## Authors' contributions

All of the authors were involved in the clinico-radiographic assessment and finalising the paper. All authors have red and approved the final version of the paper.

## Consent

Written informed consent was obtained from the parents for the purpose of publication of the manuscript and figures of their child. A copy of the written consent is available for review by the editor-in-Chief of this journal.
